# Associations of Socioeconomic Deprivation and Preterm Birth With Speech, Language, and Communication Concerns Among Children Aged 27 to 30 Months

**DOI:** 10.1001/jamanetworkopen.2019.11027

**Published:** 2019-09-11

**Authors:** Daniela Ene, Geoff Der, Sue Fletcher-Watson, Sinéad O’Carroll, Graham MacKenzie, Martin Higgins, James P. Boardman

**Affiliations:** 1 Information Services Division, NHS Lothian, NHS Scotland, Edinburgh, United Kingdom; 2 Medical Research Council/Chief Scientist Office Social and Public Health Sciences Unit, University of Glasgow, Glasgow, United Kingdom; 3 Patrick Wild Centre, University of Edinburgh, Edinburgh, United Kingdom; 4 Public Health and Health Policy, NHS Lothian, NHS Scotland, Edinburgh, United Kingdom; 5 Medical Research Council Centre for Reproductive Health, University of Edinburgh, Edinburgh, United Kingdom; 6 Centre for Clinical Brain Sciences, University of Edinburgh, Edinburgh, United Kingdom

## Abstract

**Importance:**

Successful acquisition of language is foundational for health and well-being across the life course and is patterned by medical and social determinants that operate in early life.

**Objective:**

To investigate the associations of neighborhood disadvantage, gestational age, and English as first language with speech, language, and communication concerns among children aged 27 to 30 months.

**Design, Setting, and Participants:**

This cohort study used birth data from the National Health Service maternity electronic medical record linked to the Child Health Surveillance Programme for preschool children. The cohort included 28 634 children in the United Kingdom (NHS Lothian, Scotland) born between January 2011 and December 2014 who were eligible for a health review at age 27 to 30 months between April 2013 and April 2016. Data analysis was conducted between January 2018 and February 2019.

**Exposures:**

The associations of neighborhood deprivation (using the Scottish Index of Multiple Deprivation 2016 quintiles), gestational age, and whether English was the first language spoken in the home with preschool language function were investigated using mutually adjusted logistic regression models.

**Main Outcomes and Measures:**

Speech, language, and communication (SLC) concern ascertained at age 27 to 30 months.

**Results:**

Records of 28 634 children (14 695 [51.3%] boys) with a mean (SD) age of 27.7 (2.2) months were matched. After excluding records with missing data, there were 26 341 records. The prevalence of SLC concern was 13.0% (3501 of 26 963 children with SLC data). In fully adjusted analyses, each 1-week increase in gestational age from 23 to 36 weeks was associated with an 8.8% decrease in the odds of a child having an SLC concern reported at 27 months (odds ratio, 0.92; 95% CI, 0.90-0.93). The odds of a child for whom English is not the first language of having SLC concern at age 27 to 30 months were 2.1-fold higher than those for a child whose first language is English (OR, 2.09; 95% CI, 1.66-2.64). The odds ratio for having an SLC concern among children living in the most deprived neighborhoods, compared with the least deprived neighborhoods, was 3.15 (95% CI, 2.79-3.56). The estimated probabilities for preterm children having an SLC concern were highest for those living in the most deprived areas.

**Conclusions and Relevance:**

This study found that SLC concerns at age 27 to 30 months are common and independently associated with increasing levels of neighborhood deprivation and lower gestational age. Policies that reduce childhood deprivation could be associated with improved preschool language ability and potentially avoid propagation of disadvantage across the life course, including for children born preterm.

## Key Points

**Question:**

What are the associations of socioeconomic deprivation and gestational age with preschool language ability?

**Findings:**

In this cohort study of 26 341 children in Scotland, neighborhood deprivation and lower gestational age were associated with additive risks for speech, language, and communication concerns at age 27 to 30 months.

**Meaning:**

Policies designed to lessen deprivation could be an important strategy for reducing preschool language impairment, including for children born preterm.

## Teaser

This cohort study investigates the associations of neighborhood disadvantage, gestational age, and English as a first language with speech, language, and communication concerns at age 27 to 30 months among children in Scotland.

## Introduction

Preterm birth, defined as delivery at less than 37 weeks of gestation, affects 6% to 7% of births in the United Kingdom and 10% to 11% of births worldwide.^1^ Children who are born preterm are at an increased risk of problems with language and communication that persist across childhood,^2^ and this may exert pervasive detrimental effects on life course outcomes because language skills are foundational for social-emotional development, well-being, and educational and employment outcomes.^3,4,5^

Among the general population, socioeconomic disadvantage is associated with impaired language function in childhood and with altered development of neural networks that subserve language.^6,7,8,9^ In addition, socioeconomic disadvantage is associated with preterm birth and low birth weight.^10,11,12^ Some studies suggest that preterm birth and social disadvantage confer additive risk of poor reading skills at school age, but results are inconsistent across studies,^13,14^ which may reflect geographic and temporal variation in study populations and/or variations in how disadvantage is defined. Neighborhood deprivation is modifiable and language trajectories are amenable to early intervention, so establishing the association of deprivation with language impairment in early life could offer new avenues for improving preschool abilities, including for children born preterm, whose risk of impairment is high.

The National Health Service (NHS) in Scotland offers a universal Child Health Surveillance Programme to preschool children (CHSP-PS), which is designed to identify additional support needs and promote well-being across a range of domains, including social-emotional, language, and cognitive development. Since 2013, the program has included a health review of children at age 27 to 30 months by a health professional who records a categorical outcome for speech, language, and communication (SLC) based on parental report and use of a validated tool (ie, Ages and Stages Questionnaire, Third Edition [ASQ-3]).^15^ At the same time, information about the child’s level of neighborhood deprivation from the Scottish Index of Multiple Deprivation (SIMD)^16^ is linked to the CHSP-PS using the child’s neighborhood of residence (ie, by postal code).

In this population-based study, we linked data about SLC concerns and socioeconomic deprivation from the CHSP-PS with information from maternity records. We aimed to investigate risks associated with neighborhood deprivation and gestational age on preschool language function in the general population and the associations of deprivation with preschool language function in children born preterm.

## Methods

### Sample

**Figure 1.**
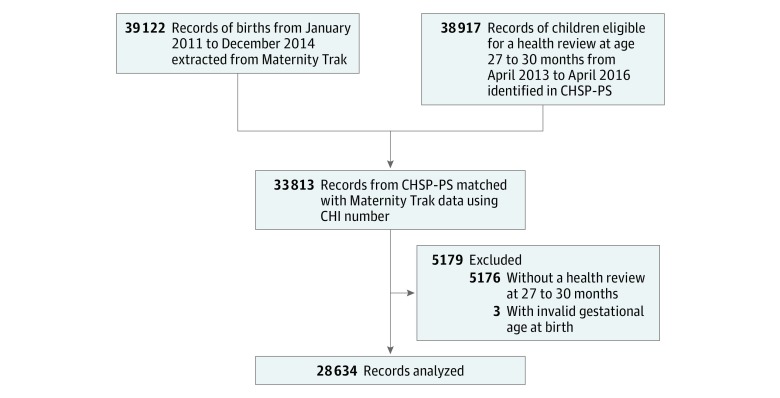
Flowchart of Data Sources and Data Matching CHSP-PS indicates Child Health Surveillance Programme–Preschool.

Figure 1 describes the derivation of the sample. The CHSP-PS system provides automated call and recall of preschool children for scheduled health reviews. We extracted data from CHSP-PS for children who were eligible for a health review at age 27 to 30 months between April 2013 and April 2016. Eligibility for review included all children aged 2 to 3 years who were registered with an NHS general practitioner and were resident in Lothian, an administrative region of Scotland with an estimated population of 889 450. The number of eligible children was 38 917.

Information from the CHSP-PS was matched with information on 39 122 births in NHS Lothian between January 2011 and December 2014. Gestational age (GA) was extracted from Maternity Trak, a system used by maternity services throughout NHS Lothian to record information about pregnancies and maternal care. The Maternity Trak extract included all women who received maternity care in NHS Lothian.

The 2 data extracts were matched using the child’s Community Health Index, which is a population register used in Scotland for health care purposes that provides a number that uniquely identifies a person on the index. Overall, 33 813 CHSP-PS records (86.9%) could be matched with the extract from Maternity Trak. Reasons for lack of matching included children born in Lothian but no longer resident when eligible for review and children who were resident in Lothian at the time of review but had been born elsewhere. Reasons for excluding data from the 33 813 matched extracts were missing health review data for age 27 to 30 months (n = 5176) and invalid GA on the birth record (n = 3).

Ethical and governance approval to link data from the CHSP-PS with GA data contained in Maternity Trak using the child’s Community Health Index number was granted by the NHS Lothian Caldicott Guardian. Data were deidentified, and informed consent was not required. This study followed the Strengthening the Reporting of Observational Studies in Epidemiology (STROBE) reporting guideline.

### Description of Measures

Gestational age was calculated from the expected date of delivery used for clinical care during the mother’s pregnancy, which was based on a first-trimester ultrasound scan. The review at age 27 to 30 months included a health visitor’s assessment of the child’s development, which was ascertained by both parental questionnaire and the ASQ-3. The ASQ-3 is a validated screening questionnaire for neurodevelopmental delay that is used in several countries for population-level assessment of early childhood development.^17,18^ The review captures preexisting and new concerns resulting from review across 9 developmental domains including SLC. For SLC, it results in a categorical outcome of no SLC concern or SLC concern, which can be preexisting or arising from the review.

The SIMD 2016 is the Scottish government’s area-based tool for identifying geographic concentrations of deprivation. Scotland is parceled into 6976 data zones, each containing approximately 760 people. The SIMD combines 7 weighted domains of deprivation as follows: (1) income; (2) employment; (3) health; (4) education, skills, and training; (5) geographic access to services; (6) crime; and (7) housing. A number of indicators are used to form a score for each domain; the 7 domain scores are then combined to form an overall SIMD ranking for each data zone (1 for most deprived to 6976 for least deprived).^16^ For the analysis here, SIMD rankings were grouped into quintiles and analyzed as a categorical variable, with quintile 1 indicating most deprived and quintile 5 indicating least deprived.

Information about GA at birth is recorded by NHS providers in Maternity Trak as completed weeks of gestation. We considered GA from 23 to 44 weeks valid. For descriptive analysis, GA was grouped into the 4 following categories: (1) 23 to 32 weeks, (2) 33 to 36 weeks, (3) 37 to 41 weeks (ie, term, the reference category), and (4) 42 to 43 weeks. In logistic regression models, GA was analyzed as a continuous variable. At the health review, the health visitor also records whether English is the first language spoken in the home, ascertained by direct question to the caregiver.

### Statistical Analysis

We used SPSS version 25 (IBM) to match the data set from CHSP-PS and Maternity Trak and for subsequent analyses. We used χ^2^ tests to investigate the unadjusted associations between the 3 independent categorical variables (ie, GA, SIMD quintile, English as first language) and the dependent variable, SLC concern.

We used 3 univariable logistic regression models to estimate the odds of having an SLC concern, using the following independent variables: model 1, GA at birth; model 2, SIMD 2016 quintile; and model 3, English as first language. Possible interactions between SIMD and GA at birth (SIMD × GA) and SIMD and English as first language spoken in the home (SIMD × English) were tested. A binomial logistic regression model that included the 3 independent variables and significant interaction terms was used to investigate associations after mutual adjustment (model 4). Results are reported as odds ratios (ORs) with 95% CIs, and a 2-tailed *P*  < .05 was considered significant. The results of the final model are displayed as a plot of fitted probabilities of an SLC concern with confidence bands. The confidence bands are calculated on the basis of 1.4 SE, in line with the recommendation of Cumming^19^ so that their separation gives a visual indication of significant differences between groups. To test for nonlinearity in gestation, we ran a logistic regression model that included the quadratic function of gestation.

## Results

### Sample Characteristics

The number of records included in the matched data set was 28 634 (14 495 [51.3%] boys; 13 939 [48.7%] girls), with a mean (SD) age of 27.7 (2.2) months. Figure 1 shows the data sources and steps taken to match the data extracts; 33 813 of 38 917 records from CHSP-PS (86.9%) were matched with Maternity Trak data. Of 33 813 matched records, 28 637 (84.7%) had a review at age 27 to 30 months and 5176 (15.3%) did not. The proportion of eligible children without review who lived in the most and least deprived areas was similar (1265 of 6516 [19.4%] in the most deprived vs 1895 of 10 936 [17.3%] in the least deprived). A total of 3 matched records had invalid GAs.

All records in the analysis data set had information about GA at birth, but 1671 (5.8%) had no information about SLC. After excluding records with missing SLC data, the prevalence of SLC concern in the sample was 13.0% (n = 3501). Overall, 818 records (2.9%) had no information about English as first language spoken in the home, and 241 (0.8%) could not be assigned an SIMD quintile owing to missing information about residency. The number of records without missing data for all 4 variables was 26 341.

### Association of GA and Socioeconomic Deprivation With SLC Concern at Age 27 to 30 Months

**Figure 2.**
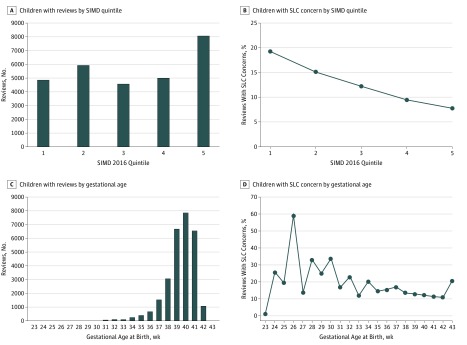
Number and Percentage of Children Reviewed With SLC Concerns Scottish Index of Multiple Deprivation (SIMD) quintile 1 indicates most deprived; SIMD quintile 5, least deprived.

Figure 2 shows the number of health reviews and the proportion associated with SLC concerns, grouped by SIMD 2016 quintile and GA. Table 1 reports the proportion of children with SLC concerns grouped by SIMD 2016 quintile, GA at birth, and English as first language. The association of SLC concern with SIMD was dose dependent across the SIMD quintile range (χ^2^_4_ = 484.004; *P* < .001) and was dose dependent with GA at birth (χ^2^_3_ = 89.863; *P* < .001). The association of English not being the first language spoken in the home with SLC concern was also significant (χ^2^_1_ = 64.109; *P* < .001).

**Table 1.** Number and Percentage of Children With SLC Concerns by SIMD 2016 Quintile, GA at Birth, and English as First Language

Variable	No. (%)			
	SLC Concern (n = 3501)^a^	No SLC Concern (n = 23 462)^a^	Missing SLC Information (n = 1671)^a^	No. of Reviews (N = 28 634)^b^
SIMD 2016 quintile^c^				
1	934 (19.2)	3536 (72.7)	393 (8.1)	4863 (17.0)
2	895 (15.1)	4712 (79.5)	321 (5.3)	5928 (20.7)
3	559 (12.2)	3779 (82.4)	247 (5.4)	4585 (16.0)
4	464 (9.4)	4234 (85.7)	242 (4.9)	4940 (17.3)
5, Reference category	627 (7.8)	6999 (86.7)	451 (5.6)	8077 (28.2)
Missing or unmatched postal code	22 (9.1)	202 (83.8)	17 (7.1)	241 (0.8)
GA, wk				
23-32	85 (23.9)	201 (57.1)	66 (18.8)	352 (1.2)
33-36	227 (14.9)	1177 (77.2)	121 (7.9)	1525 (5.3)
37-41, Reference category	3081 (12.0)	21 184 (82.4)	1444 (5.6)	25 709 (89.8)
42-43	108 (10.3)	900 (85.9)	40 (3.8)	1048 (3.7)
English as first language				
No	596 (16.3)	2845 (77.9)	210 (5.8)	3651 (12.8)
Yes, reference category	2866 (11.9)	20 256 (83.8)	1043 (4.3)	24 165 (84.4)
Missing or incomplete information	39 (4.8)	361 (44.1)	418 (51.1)	818 (2.9)

Abbreviations: GA, gestational age; SIMD, Scottish Index of Multiple Deprivation; SLC, speech, language, and communication.

^a^ Proportion of number of reviews per category of SIMD quintile, grouped GA, and English as first language.

^b^ Proportion of total number of reviews (N = 28 634).

^c^ First quintile indicates most deprived; fifth quintile, least deprived.

Table 2 shows univariable and binomial logistic regression models used to investigate associations of SIMD quintile, GA, and English as first language with SLC concern at age 27 to 30 months. In model 4, for each 1-week increase in GA at birth, there was an 8.8% decrease in the odds of a child having SLC concerns at the health review at age 27 to 30 months (OR, 0.92; 95% CI, 0.90-0.93). The odds of a child having SLC concerns at age 27 to 30 months were 3.2-fold higher if the child lives in the most deprived quintile compared with a child living in the least deprived quintile (OR, 3.15; 95% CI, 2.79-3.56), with a progressive reduction of risk from most to least deprived quintiles. The odds of a child for whom English is not the first language of having SLC concern at age 27 to 30 months were 2.1-fold higher than those for a child whose first language is English (OR, 2.09; 95% CI, 1.66-2.64).

**Table 2.** Logistic Regression Models for Likelihood of Speech, Language, and Communication Concerns at Age 27 to 30 Months

Factor	Odds Ratio (95% CI)				*P* Value
	Model 1	Model 2	Model 3	Model 4	
GA	0.91 (0.90-0.93)	NA	NA	NA	<.001
SIMD 2016 quintile^a^					
1	NA	2.95 (2.64-3.29)	NA	NA	<.001
2	NA	2.12 (1.90-2.36)	NA	NA	<.001
3	NA	1.65 (1.46-1.86)	NA	NA	<.001
4	NA	1.22 (1.08-1.39)	NA	NA	<.001
5	NA	1 (Reference)	NA	NA	<.001
English not first language	NA	NA	1.48 (1.34-1.63)	NA	<.001
GA	NA	NA	NA	0.92 (0.90-0.93)	<.001
SIMD 2016 quintile^a^					
1	NA	NA	NA	3.15 (2.79-3.56)	<.001
2	NA	NA	NA	2.25 (1.99-2.53)	<.001
3	NA	NA	NA	1.75 (1.54-2.00)	<.001
4	NA	NA	NA	1.25 (1.09-1.43)	<.001
5	NA	NA	NA	1 (Reference)	<.001
English not first language	NA	NA	NA	2.09 (1.66-2.64)	<.001
English first language × SIMD 2016 quintile^a^					<.001
1	NA	NA	NA	0.49 (0.37-0.66)	<.001
2	NA	NA	NA	0.56 (0.41-0.76)	<.001
3	NA	NA	NA	0.59 (0.42-0.83)	<.001
4	NA	NA	NA	0.84 (0.59-1.19)	.33
5	NA	NA	NA	1 (Reference)	NA

Abbreviations: GA, gestational age; NA, not applicable; SIMD, Scottish Index of Multiple Deprivation.

^a^ First quintile indicates most deprived; fifth quintile, least deprived.

There was significant interaction between SIMD and English as first language spoken in the home (Table 2), but the interaction term between GA and SIMD quintile was not significant (eTable in the Supplement). The quadratic function of gestation was not significant in the model.

**Figure 3.**
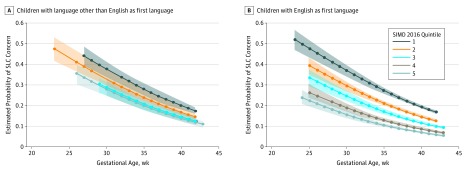
Estimated Probability of Having SLC Concerns Shaded areas indicate confidence bands at 1.4 × SE. Scottish Index of Multiple Deprivation (SIMD) quintile 1 indicates most deprived; SIMD quintile 5, least deprived.

Figure 3 shows the estimated probabilities of having SLC concern at age 27 to 30 months were higher for those with lower GA and highest for children with low GA who lived in the most deprived areas compared with those with low GA who lived in the least deprived areas.

## Discussion

By linking information from a national child health surveillance program to the maternity record for 26 341 children, we found that neighborhood deprivation and lower GA were independently associated with SLC concern at age 27 to 30 months. The data suggest that preterm birth and neighborhood deprivation interact additively to produce greater risk among children with both exposures; the risk was dose dependent with increasing levels of deprivation and lower GA.

These findings are consistent with the theory that socioeconomic disadvantage in childhood shapes neurodevelopmental and health outcomes across the life course.^20^ Our findings suggest that the association of language with socioeconomic status^21^ is apparent during preschool years and can be ascertained through routinely collected data at the population level. We chose to characterize socioeconomic disadvantage using the SIMD because neighborhood deprivation is consistently associated with health inequality, including adverse birth outcomes,^12^ it encompasses 7 features of deprivation relevant to the population we studied, and it is collected routinely by the Scottish government, so has utility for assessing the impact of policy change.

Our data indicate that neighborhood deprivation is associated with adverse preschool language development. These findings are consistent with observations that different dimensions of poverty that operate in the perinatal period—biological, psychosocial, and social or infrastructural corisks that often accompany monetary poverty—are associated with language impairment.^22^ For example, maternal substance use,^23^ low breastfeeding rates,^24^ maternal depression,^25^ and reduced access to preschool education^26^ are all associated with language impairment at school age. Plausible mechanisms that explain the association of deprivation with adverse neurodevelopmental outcomes include gestational immune dysregulation,^27^ alterations to the maternal hypothalamic-pituitary-adrenal axis that arise in situations of prenatal environmental stress and are associated with adverse behavioral outcomes in the offspring,^28,29^ and epigenomic variation associated with environmental adversity in pregnancy.^30,31^

Several studies report that children born preterm have increased susceptibility to language deficits manifest during infancy and childhood. These include problems with social communication and symbolic skills, vocabulary, semantics, morphological and syntactic complexity, verbal processing speed and memory, and reading.^2,32,33,34,35,36^ Further work is required to determine whether having an SLC concern at the health review at age 27 to 30 months reflects these language difficulties or whether it reflects other cognitive, behavioral, or sensory problems that are prevalent among the preterm population and can coexist with language difficulties. The observation that not having English as the first language spoken at home was associated with SLC concern may reflect the use of an English language assessment tool or could be because the variable is a proxy indicator of racial or immigrant status.

### Applicability to Clinical and Education Practice

Language skills are critical for socioemotional development, well-being, and educational and employment outcomes.^4,5^ In the United Kingdom, child poverty has increased year on year since 2011 to 2012; more than 4 million children—1 of 3—now live in poverty.^37^ Our observation of an association of SIMD quintile with SLC concern suggests that policies designed to tackle rising rates of child poverty could reduce the burden of preschool language difficulties and, consequently, improve important life outcomes. Deprivation and GA were additive in mutually adjusted regression models, which implies that reducing deprivation could impact language outcomes, including among children born preterm. There was a dose-dependent increase in prevalence of SLC concern with younger GA. This pattern mirrors that reported for reduced intelligence quotient^38^ and the need for special educational support at school age^39^ among children and adolescents born preterm.

Speech, language, and communication concerns captured routinely as part of the child health surveillance program may be an effective method for identifying children at risk of impairment during preschool years. This is important because follow-up programs for preterm infants in the United Kingdom and other countries do not routinely extend beyond 2 years corrected age, which means that language difficulties may not be detected until the child enters school. Use of population-level screening for early identification of difficulty provides an opportunity for early intervention in a group of children who are at high risk for impairment.

### Strengths and Limitations

A major strength of our study was its large size, with high-quality, detailed maternity and childhood records and record linkage rate of 87%. Gestational age was calculated from the expected date of delivery used for clinical care during the mother’s pregnancy and based on first-trimester ultrasound results, and the SIMD is defined by postal code of residence. Therefore neither GA nor neighborhood deprivation relied on self-report, which can be imprecise for both measurements. The ASQ-3 is a validated screening tool that can be used by parents or practitioners and is used in real-world, population-level assessments in several countries. Although there was an association of children growing up in a household where English was not the first language spoken in the home with risk of SLC concern at age 27 to 30 months, we adjusted for this in analyses and reported similar associations of deprivation and preterm birth for this population of children. A further strength of our study was that we had long enough follow-up for language problems to manifest. However, the population was still quite young, so our study might have underestimated the size of the association. Further follow-up of the cohort of children as it ages could help determine whether SLC concern at age 27 to 30 months is sustained or whether it reflects transient developmental delay.

Our study has some limitations. We cannot separate the direct associations of childhood neighborhood deprivation from shared parental determinants associated with living in deprived neighborhoods that might influence preschool language abilities. For example, neighborhood deprivation overlaps with low neighborhood adult education, intellectual ability is heritable and is socially patterned, and maternal mental health problems cluster in families living in deprived situations. Nor can we separate the associations of GA from the comorbidities of preterm birth that influence cognitive development.^14^ Nevertheless, our results suggest that children living in neighborhood deprivation, especially those born preterm, belong to a high-risk group that should be assessed for language ability. If appropriate speech and language therapeutic interventions were put in place in early childhood for those identified to be at risk, propagation of disadvantage across the life course could potentially be avoided.

## Conclusions

Our data build on the literature showing an association of socioeconomic disadvantage with adverse childhood outcomes. They show an additive risk of neighborhood deprivation and preterm birth associated with preschool language difficulties. These findings highlight the urgent need for strategies to reduce the number of children growing up in deprived situations and the need to assess children who were born preterm for difficulties with SLC during the preschool years.

## Notes

Supplement. **eTable.** Binomial Logistic Regression for SLC Concern at Health Review at Age 27 to 30 Months, Including Interaction Between Gestational Age and Deprivation Quintile
