# Supplementary material for: Associations of Socioeconomic Deprivation and Preterm Birth With Speech, Language, and Communication Concerns Among Children Aged 27 to 30 Months
Source: JAMA Netw Open. 2019 Sep 11;2(9):e1911027. doi: 10.1001/jamanetworkopen.2019.11027 (PMC6739726; doi:10.1001/jamanetworkopen.2019.11027)
Supplement: Supplement. — eTable. Binomial Logistic Regression for SLC Concern at Health Review at Age 27 to 30 Months, Including Interaction Between Gestational Age and Deprivation Quintile [file jamanetwopen-2-e1911027-s001.pdf]

Supplementary Online Content

Ene D, Der G, Fletcher-Watson S, et al. Association of socioeconomic deprivation and preterm birth with speech, language, and communication concerns among children aged 27 to 30 months. *JAMA Netw Open*. 2019;2(9):e1911027. doi:10.1001/jamanetworkopen.2019.11027

eTable. Binomial Logistic Regression for SLC Concern at Health Review at Age 27 to 30 Months, Including Interaction Between Gestational Age and Deprivation Quintile

This supplementary material has been provided by the authors to give readers additional information about their work.

© 2019 Ene D et al. *JAMA Network Open.*

eTable. Binomial Logistic Regression for SLC Concern at Health Review at Age 27 to 30 Months, Including Interaction Between Gestational Age and Deprivation Quintile

Variable	OR	95% CI for Odds Ratio		p-value
		lower	upper	
SIMD 2016 Quintile 1 (most deprived)	1·102	0·158	7·687	0·92
SIMD 2016 Quintile 2	0·301	0·042	2·172	0·23
SIMD 2016 Quintile 3	0·349	0·038	3·224	0·35
SIMD 2016 Quintile 4	0·293	0·028	3·107	0·31
SIMD 2016 Quintile (least deprived)				0·53
GA	0·889	0·857	0·922	<0·001
English as First Language = No	1·306	1·183	1·442	<0·001
GA x SIMD Quintile 1 (most deprived)	1·024	0·975	1·076	0·35
GA x SIMD Quintile 2	1·051	0·999	1·105	0·06
GA x SIMD Quintile 3	1·040	0·983	1·101	0·17
GA x SIMD Quintile 4	1·037	0·977	1·102	0·24
GA x SIMD Quintile 5 (least deprived)				0·39

© 2019 Ene D et al. *JAMA Network Open.*
